# Impact of Alcohol Intake on Body Composition in Patients with Steatotic Liver Disease

**DOI:** 10.3390/nu17061092

**Published:** 2025-03-20

**Authors:** Masahiro Matsui, Akira Fukuda, Saori Onishi, Kosuke Ushiro, Tomohiro Nishikawa, Akira Asai, Soo Ki Kim, Hiroki Nishikawa

**Affiliations:** 1 Second Department of Internal Medicine, Osaka Medical and Pharmaceutical University, Takatsuki 569-8686, Osaka, Japan; 2 Osaka Medical and Pharmaceutical University Health Science Clinic, Takatsuki 569-8686, Osaka, Japan; 3 Department of Gastroenterology, Kobe Asahi Hospital, Kobe 653-8501, Hyogo, Japan

**Keywords:** steatotic liver disease, alcohol, fat-free mass, fat mass

## Abstract

**Objectives:** To examine the effect of alcohol intake on body composition in patients with steatotic liver disease (SLD). **Methods**: In men, group A (n = 819) was defined as non-drinkers, group B (n = 1147) as <30 g of ethanol equivalent per day, group C (n = 125) as between 30 and 60 g/day, and group D (n = 344) as >60 g/day. In women, group A (n = 749) was defined as non-drinkers, group B (n = 354) as <20 g/day, group C (n = 36) as between 20 and 50 g/day, and group D (n = 68) as >50 g/day. The fat-free (FF) index and fat (F) index were defined as FF mass and F mass divided by height squared (kg/m^2^). Results: The average FF index and F index in groups A, B, C, and D in men were 19.01, 19.29, 18.50, and 18.55 kg/m^2^ (overall *p* < 0.0001), and 6.28, 6.71, 5.66, and 6.03 kg/m^2^ (overall *p* < 0.0001). The average FF index and F index in groups A, B, C, and D in women were 16.03, 15.96, 15.62, and 15.07 kg/m^2^ (overall *p* < 0.0001), and 9.89, 9.02, 9.32, and 7.53 kg/m^2^ (overall *p* < 0.0001). **Conclusions**: Heavy drinking has a negative effect on skeletal muscle and fat, but complete abstinence from alcohol may not be necessary in SLD patients.

## 1. Introduction

In Japan, alcohol has been consumed at dinners, festivals, and many other occasions for a long time and has become a familiar part of daily life [1]. However, alcohol consumption involves a lot of problems for health maintenance. In the country, alcohol consumption has been declining with the decrease in the total population and aging, and the proportion of male drinkers has slightly declined while the proportion of heavy drinkers has been slightly increasing [2]. In Japan, there are more than 10 million habitual drinkers with an increased risk of lifestyle-related diseases and more than 1 million alcohol-dependent patients [3]. Excessive alcohol intake can be a risk factor for various diseases, such as cardiovascular diseases [4], metabolic diseases [5], liver diseases [6], and malignancies [7,8], and can be associated with poorer prognoses [9]. The Japan Society of Hepatology has reported that although hepatitis C used to be the leading cause of cirrhosis, in recent years, alcoholic causes have overtaken hepatitis C as the leading cause [10].

The impact of alcohol on skeletal muscle is a relatively recent topic [11]. Heavy drinking has adverse effects on skeletal muscle due to direct damage to skeletal muscle caused by acetaldehyde [12], decreased ammonia clearance [13], increased intestinal permeability [14], decreased food intake other than alcohol (i.e., starvation) [15], gonadal hormone abnormalities [16,17], and growth hormone abnormalities [17]. A large study from the United Kingdom (n = 12,294) reported that skeletal muscle mass decreases with increasing alcohol intake [18]. On the other hand, acetaldehyde, a metabolite of ethanol, inhibits lipolysis [19]. Triglyceride (TG) is a lipid that is greatly affected by alcohol consumption, and TGs that remain in the liver accumulate in the liver and cause steatotic liver disease (SLD) [20]. SLD is characterized by fat deposition of more than 20% in the liver. Ultrasonography (US) is often the trigger for diagnosis, but computed tomography or magnetic resonance imaging can also be used to diagnose SLD [21]. Metabolic dysfunction-associated steatotic liver disease (MASLD) is a condition that combines metabolic abnormalities in addition to SLD and has received considerable attention in recent years [21,22]. MASLD is defined as an alcohol intake of less than 210 g per week for men and 140 g per week for women, and if alcohol intake is greater than that, it is named metabolic-alcoholic liver disease (met-ALD) or ALD [21]. However, reports examining the effects of alcohol on body composition, such as muscle mass and fat mass, in patients with SLD are rare to our knowledge. To clarify these clinical research questions may be of importance.

The purpose of this study was therefore to examine the effect of alcohol intake on body composition in patients with SLD.

## 2. Patients and Methods

### 2.1. Patients and Our Study

A total of 3642 individual SLD cases with available data on body composition and alcohol intake were confirmed in our medical records between October 2021 and November 2023 and were analyzed in a retrospective manner. In all analyzed cases, SLD was confirmed through US. The amount of alcohol consumption was ascertained by means of a questionnaire. All study subjects were tested at the Osaka Medical and Pharmaceutical University (OMPU) Health Sciences Clinic (OMPU-attached facility). The procedure of our body composition measurement was as noted before [23]. Fat-free (FF) mass (kg) and fat (F) mass were measured and analyzed in the current analysis. The FF index was defined as FF mass divided by height squared (kg/m^2^). The F index was defined as fat mass divided by height squared (kg/m^2^). The fatty liver index (FLI) was calculated using the body mass index (BMI), TG, gamma glutamyl transferase, and waist circumference (WC), as reported elsewhere [24]. The severity of SLD was divided into the three categories (mild, moderate, and severe SLD), considering US findings (i.e., hepatorenal echo contrast, intrahepatic vascular blurring, and deep attenuation) [25].

### 2.2. Group Classification Based on Alcohol Intake

In men, group A was defined as non-drinkers, group B as those who drank <210 g of ethanol equivalent per week (<30 g per day), group C as those who drank between 210 g and 420 g of ethanol equivalent per week (30–60 g per day), and group D as those who drank >420 g of ethanol equivalent per week (>60 g per day) [21]. In women, group A was defined as non-drinkers, group B as those who drank <140 g of ethanol equivalent per week (<20 g per day), group C as those who drank between 140 g and 350 g of ethanol equivalent per week (20–50 g per day), and group D as those who drank >350 g of ethanol equivalent per week (>50 g per day) [21].

We compared the FF index, F index, and other baseline features among groups A, B, C, and D. This study conformed to the ethical guidelines of the Declaration of Helsinki and was approved by the ethics committee of OMPU hospital (approval no. 2023-159). This study was exempted from the requirement of written informed consent due to the retrospective nature of the study. All data were analyzed anonymously.

### 2.3. Statistics

In the two-group comparison (continuous variables), an unpaired *t* test or Mann-Whitney *U*-test was applied, as appropriate, after verifying equal dispersity. In the multiple-group comparison (continuous variables), analysis of variance (ANOVA) or the Kruskal-Wallis test was applied, as appropriate, after verifying equal dispersity. In the group comparison (nominal variables), Fisher’s exact test was applied. Unless otherwise stated, data were shown as a number or average (±standard deviation (SD)). A *p* value less than 0.05 was considered to indicate a statistically significant difference. JMP 17.0.0 software (SAS Institute, Cary, NC, USA) was applied to perform statistical analyses.

## 3. Results

### 3.1. Baseline Features

Baseline features in the present analyses are shown in Table 1. The average (±SD) age in men (n = 2435) and women (n = 1207) was 55.3 ± 10.9 years and 56.8 ± 10.1 years (*p* < 0.0001). The average (±SD) BMI in men and women was 25.5 ± 3.5 kg/m^2^ and 25.4 ± 4.0 kg/m^2^ (*p* = 0.2110). The proportion of cases with groups A, B, C, and D was 33.6% (819/2435), 47.1% (1147/2435), 5.1% (125/2435), and 14.1% (344/2435) in men, and 62.1% (749/1207), 29.3% (354/1207), 3.0% (36/1207), and 5.6% (68/1207) in women (*p* < 0.0001). The proportion of cases with mild, moderate, and severe SLD through US was 47.2% (1150/2435), 40.0% (975/2435), and 12.7% (310/2435) in men, and 53.3% (643/1207), 36.2% (437/1207), and 10.5% (127/1207) in women (*p* = 0.0021). The average (±SD) WC in men and women was 90.2 ± 8.9 cm and 88.2 ± 9.6 cm (*p* < 0.0001). The average (±SD) FIB4 index in men and women was 1.15 ± 0.58 and 1.11 ± 0.48 (*p* = 0.5322). The average (±SD) FF index in men and women was 19.0 ± 1.4 kg/m^2^ and 15.9 ± 0.9 kg/m^2^ (*p* < 0.0001). The average (±SD) F index in men and women was 6.4 ± 2.3 kg/m^2^ and 9.5 ± 3.2 kg/m^2^ (*p* < 0.0001).

### 3.2. Comparison of FF Index and F Index Between Four Groups in Men

**Figure 1:**
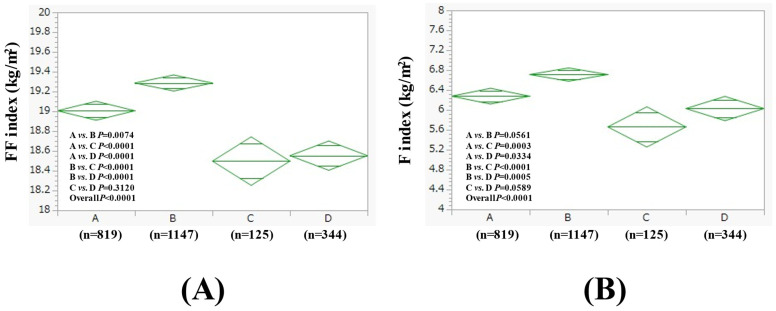
FF index (**A**) and F index (**B**) among four groups in men (n = 2435). Group A was defined as non-drinkers, group B as those who drank less than 30 g of ethanol equivalent per day, group C as those who drank between 30–60 g of ethanol equivalent per day, and group D as those who drank more than 60 g of ethanol equivalent per day.

The average (±SD) FF index in groups A, B, C, and D in men was 19.01 ± 1.17 kg/m^2^, 19.29 ± 1.56 kg/m^2^, 18.50 ± 0.94 kg/m^2^, and 18.55 ± 1.47 kg/m^2^ (*p* values (two-group comparison): A vs. B, *p* = 0.0074; A vs. C, *p* < 0.0001; A vs. D, *p* < 0.0001; B vs. C, *p* < 0.0001; B vs. D, *p* < 0.0001; C vs. D, *p* = 0.3120. Overall *p* < 0.0001) (Figure 1A). The average (±SD) F index in groups A, B, C, and D in men was 6.28 ± 1.88 kg/m^2^, 6.71 ± 2.73 kg/m^2^, 5.66 ± 1.50 kg/m^2^, and 6.03 ± 1.89 kg/m^2^ (*p* values (two-group comparison): A vs. B, *p* = 0.0561; A vs. C, *p* = 0.0003; A vs. D, *p* = 0.0334; B vs. C, *p* < 0.0001; B vs. D, *p* = 0.0005; C vs. D, *p* = 0.0589. Overall *p* < 0.0001) (Figure 1B). The results for the comparison of other baseline data in addition to the FF index and F index among four groups in men are listed in Table 2.

### 3.3. Comparison of FF Index and F Index Between Four Groups in Women

**Figure 2:**
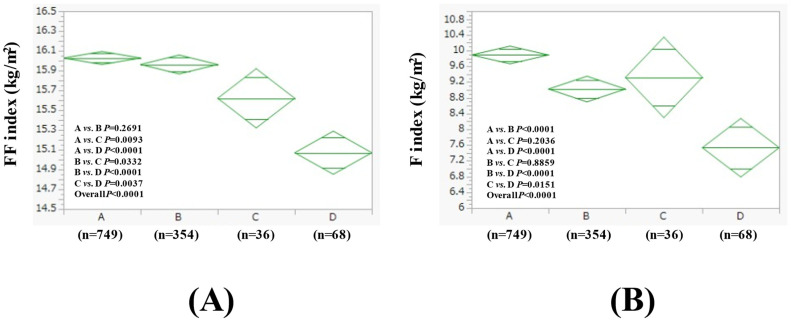
FF index (**A**) and F index (**B**) among four groups in women (n = 1207). Group A was defined as non-drinkers, group B as those who drank less than 20 g of ethanol equivalent per day, group C as those who drank between 20–50 g of ethanol equivalent per day, and group D as those who drank more than 50 g of ethanol equivalent per day.

The average (±SD) FF index in groups A, B, C, and D in women was 16.03 ± 0.93 kg/m^2^, 15.96 ± 0.85 kg/m^2^, 15.62 ± 1.06 kg/m^2^, and 15.07 ± 1.01 kg/m^2^ (*p* values (two-group comparison): A vs. B, *p* = 0.2691; A vs. C, *p* = 0.0093; A vs. D, *p* < 0.0001; B vs. C, *p* = 0.0332; B vs. D, *p* < 0.0001; C vs. D, *p* = 0.0037. Overall *p* < 0.0001) (Figure 2A). The average (±SD) F index in groups A, B, C, and D in men was 9.89 ± 3.24 kg/m^2^, 9.02 ± 2.83 kg/m^2^, 9.32 ± 3.72 kg/m^2^, and 7.53 ± 2.90 kg/m^2^ (*p* values (two-group comparison): A vs. B, *p* < 0.0001; A vs. C, *p* = 0.2036; A vs. D, *p* < 0.0001; B vs. C, *p* = 0.8859; B vs. D, *p* < 0.0001; C vs. D, *p* = 0.0151. Overall *p* < 0.0001) (Figure 2B). The results for the comparison of other baseline data in addition to the FF index and F index among four groups in women are listed in Table 3.

### 3.4. Subgroup Analysis 1: Comparison of FF Index and F Index Between Four Groups in Men with BMI > 25 kg/m^2^ and BMI ≤ 25 kg/m^2^

**Figure 3:**
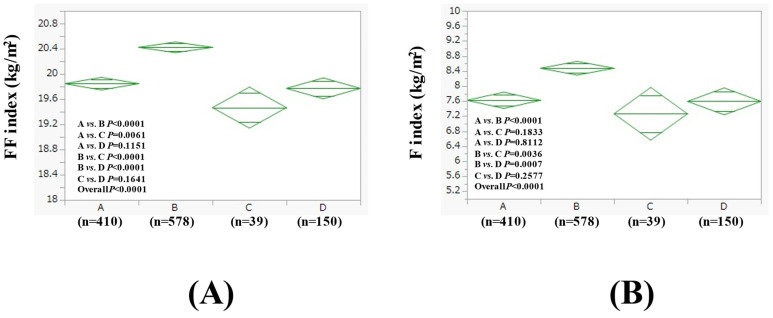
FF index (**A**) and F index (**B**) among four groups in men with BMI > 25 kg/m^2^ (n = 1177).

We conducted subgroup analyses based on the baseline BMI value. The average (±SD) FF index in groups A (n = 410), B (n = 578), C (n = 39), and D (n = 150) in men with BMI > 25 kg/m^2^ (n = 1177) was 19.85 ± 0.79 kg/m^2^, 20.42 ± 1.24 kg/m^2^, 19.47 ± 0.60 kg/m^2^, and 19.77 ± 0.85 kg/m^2^ (*p* values (two-group comparison): A vs. B, *p* < 0.0001; A vs. C, *p* = 0.0061; A vs. D, *p* = 0.1151; B vs. C, *p* < 0.0001; B vs. D, *p* < 0.0001; C vs. D, *p* = 0.1641. Overall *p* < 0.0001) (Figure 3A). The average (±SD) F index in groups A, B, C, and D in men with BMI > 25 kg/m^2^ was 7.62 ± 1.60 kg/m^2^, 8.48 ± 2.75 kg/m^2^, 7.26 ± 1.31 kg/m^2^, and 7.59 ± 1.61 kg/m^2^ (*p* values (two-group comparison): A vs. B, *p* < 0.0001; A vs. C, *p* = 0.1833; A vs. D, *p* = 0.8112; B vs. C, *p* = 0.0036; B vs. D, *p* = 0.0007; C vs. D, *p* = 0.2577. Overall *p* < 0.0001) (Figure 3B).

**Figure 4:**
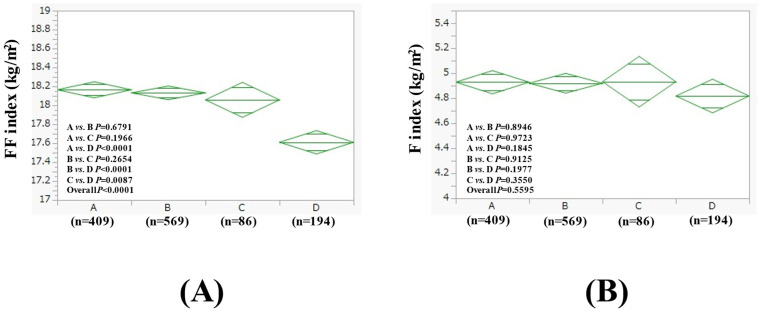
FF index (**A**) and F index (**B**) among four groups in men with BMI ≤ 25 kg/m^2^ (n = 1258).

The average (±SD) FF index in groups A (n = 409), B (n = 569), C (n = 86), and D (n = 194) in men with BMI ≤ 25 kg/m^2^ (n = 1258) was 18.17 ± 0.84 kg/m^2^, 18.13 ± 0.83 kg/m^2^, 18.06 ± 0.70 kg/m^2^, and 17.61 ± 1.12 kg/m^2^ (*p* values (two-group comparison): A vs. B, *p* = 0.6791; A vs. C, *p* = 0.1966; A vs. D, *p* < 0.0001; B vs. C, *p* = 0.2654; B vs. D, *p* < 0.0001; C vs. D, *p* = 0.0087. Overall *p* < 0.0001) (Figure 4A). The average (±SD) F index in groups A, B, C, and D in men with BMI ≤ 25 kg/m^2^ was 4.93 ± 0.92 kg/m^2^, 4.92 ± 0.98 kg/m^2^, 4.93 ± 0.89 kg/m^2^, and 4.82 ± 0.99 kg/m^2^ (*p* values (two-group comparison): A vs. B, *p* = 0.8946; A vs. C, *p* = 0.9723; A vs. D, *p* = 0.1845; B vs. C, *p* = 0.9125; B vs. D, *p* = 0.1977; C vs. D, *p* = 0.3550. Overall *p* = 0.5595) (Figure 4B).

### 3.5. Subgroup Analysis 2: Comparison of FF Index and F Index Among Four Groups in Women with BMI > 25 kg/m^2^ and BMI ≤ 25 kg/m^2^

**Figure 5:**
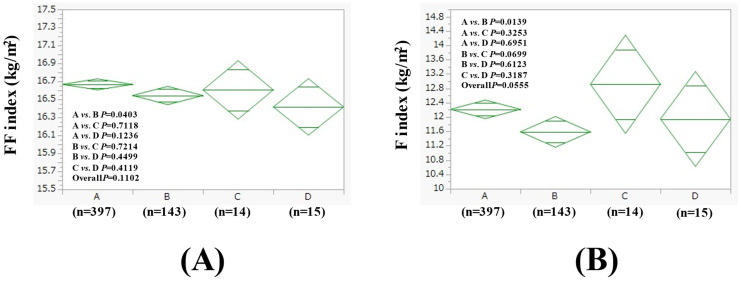
FF index (**A**) and F index (**B**) among four groups in women with BMI > 25 kg/m^2^ (n = 569).

The average (±SD) FF index in groups A (n = 397), B (n = 143), C (n = 14), and D (n = 15) in women with BMI > 25 kg/m^2^ (n = 569) was 16.67 ± 0.60 kg/m^2^, 16.54 ± 0.64 kg/m^2^, 16.61 ± 0.71 kg/m^2^, and 16.42 ± 0.91 kg/m^2^ (*p* values (two-group comparison): A vs. B, *p* = 0.0403; A vs. C, *p* = 0.7118; A vs. D, *p* = 0.1236; B vs. C, *p* = 0.7214; B vs. D, *p* = 0.4499; C vs. D, *p* = 0.4119. Overall *p* = 0.1102) (Figure 5A). The average (±SD) F index in groups A, B, C, and D in women with BMI > 25 kg/m^2^ was 12.22 ± 2.62 kg/m^2^, 11.59 ± 2.57 kg/m^2^, 12.91 ± 3.15 kg/m^2^, and 11.95 ± 2.19 kg/m^2^ (*p* values (two-group comparison): A vs. B, *p* = 0.0139; A vs. C, *p* = 0.3253; A vs. D, *p* = 0.6951; B vs. C, *p* = 0.0699; B vs. D, *p* = 0.6123; C vs. D, *p* = 0.3187. Overall *p* = 0.0555) (Figure 5B).

**Figure 6:**
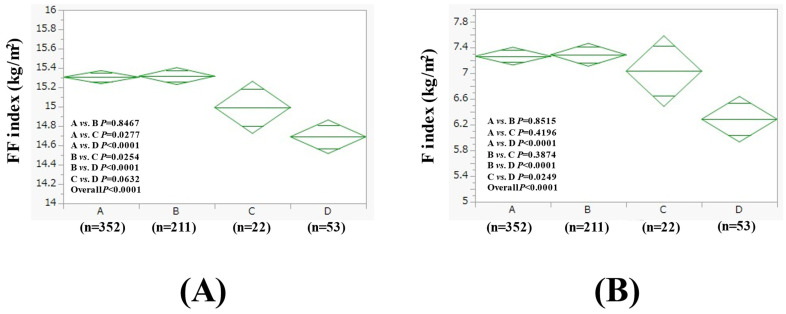
FF index (**A**) and F index (**B**) among four groups in women with BMI ≤ 25 kg/m^2^ (n = 638).

The average (±SD) FF index in groups A (n = 352), B (n = 211), C (n = 22), and D (n = 53) in women with BMI ≤ 25 kg/m^2^ (n = 638) was 15.31 ± 0.67 kg/m^2^, 15.32 ± 0.58 kg/m^2^, 14.99 ± 0.70 kg/m^2^, and 14.69 ± 0.66 kg/m^2^ (*p* values (two-group comparison): A vs. B, *p* = 0.8467; A vs. C, *p* = 0.0277; A vs. D, *p* < 0.0001; B vs. C, *p* = 0.0254; B vs. D, *p* < 0.0001; C vs. D, *p* = 0.0632. Overall *p* < 0.0001) (Figure 6A). The average (±SD) F index in groups A, B, C, and D in women with BMI ≤ 25 kg/m^2^ was 7.27 ± 1.30 kg/m^2^, 7.29 ± 1.23 kg/m^2^, 7.03 ± 1.69 kg/m^2^, and 6.29 ± 1.53 kg/m^2^ (*p* values (two-group comparison): A vs. B, *p* = 0.8515; A vs. C, *p* = 0.4196; A vs. D, *p* < 0.0001; B vs. C, *p* = 0.3874; B vs. D, *p* < 0.0001; C vs. D, *p* = 0.0249. Overall *p* < 0.0001) (Figure 6B).

## 4. Discussion

SLD patients account for about one out of every four people in Japan [26]. In the present study, we examined the effects of alcohol on SLD patients from the perspective of body composition. Alcohol causes various diseases [27]. In addition, since body composition is closely correlated with prognosis [28], the results of this study are highly significant. The framework of this study was to examine the extent to which alcohol consumption is acceptable in patients with SLD from the perspective of body composition. In this study, we observed a marked difference in the number of cases between men and women, with 2435 cases in men and 1207 cases in women, which may be partly due to the higher prevalence of SLD in men than in women in our country [29].

In the overall analysis, group B had the highest FF index and F index among men, while groups C and D had the lowest FF index and F index. Among women, the FF index was not significantly different between groups A and B, with group D having the lowest value, and the F index was the highest in group A and the lowest in group D. These study results suggested that, from the perspective of skeletal muscle mass, excessive alcohol consumption is associated with a decrease in skeletal muscle mass, but not to the extent that it is necessary to completely abstain from alcohol consumption. Small amounts of alcohol consumption have been reported to slightly increase skeletal muscle mass [18]. Excessive alcohol consumption increases myostatin, a myokine that inhibits muscle protein synthesis [30]. Many previous studies have shown that skeletal muscle mass is a prognostic factor in patients with SLD [31], and the present results are considered to involve important findings for clinicians. On the other hand, women are generally more inclined to store a significant amount of fat from fat reserves and generate energy rather than from skeletal muscle reserves, which may make women more resistant to skeletal muscle wasting compared to men [32]. The present results on fat mass are characterized by a decrease in the F index, especially in women in group D. Women in group D had a lower BMI and WC, presumably due to a decrease in both the FF index and F index associated with lower dietary intake. The FLI was the highest in men in group D, possibly due to alcoholic SLD, but in women, FLI tended to be rather lower in group D compared to groups A, B, and C. Similarly, lower dietary intake may be a contributing factor to these results. Nutritional guidance may be necessary for such high-risk patients.

In this study, 51.7% (1258/2435) of men and 52.9% (638/1207) of women had non-obese SLD with a BMI below 25 kg/m^2^. Ye et al. reported that in the SLD population, 19.2% were lean and 40.8% were non-obese, which was lower than our data [33]. Differences in PNPLA3 gene polymorphisms may have an impact [34]. As shown in Figure 4, it should be noted that in non-obese male SLD, the effect on skeletal muscle mass was greater than on fat in group D. In contrast, as shown in Figure 6, group D had a strong effect on both fat mass and skeletal muscle mass in non-obese female SLD. In Japan, the number of drinkers at risk of health problems has been increasing, especially among women [2], and caution is needed. It has also been reported that myostatin is closely related to hepatic fat accumulation in non-obese SLD [35].

The Japanese Ministry of Health, Labor, and Welfare states that the amount that increases the risk of lifestyle-related diseases is a daily net alcohol intake of 40 g or more for men and 20 g or more for women [2]. In our data, for men, groups A and B accounted for 80.7% (1966/2435) of the total; for women, groups A and B accounted for 91.4% (1103/1207) of the total. Thus, in our study cohort, the majority of drinkers are below the drinking level of health risk, but cases with higher drinking levels require firm interventions such as brief intervention [36,37,38] and drug intervention [39,40,41]. The concept of “harm reduction” through reduced alcohol consumption has been proposed in recent years [42].

A note must be made of the limitations of the present study. One is that this study involved a retrospective, single-center, and cross-sectional study. Another is that the study was limited to Japanese SLD patients, so it is unknown whether the same is true for other racial populations, such as Europeans. The standard value of muscle mass is different between Westerners and Japanese because of the difference in their physiques. Additionally, the amount of daily alcohol intake was calculated based on self-reports, and body composition can be greatly affected by the amount of exercise and diet, as well as the number of cases in groups C or D, which was relatively smaller compared to the number of cases in groups A or B. Baseline features among groups A, B, C, and D were also largely different. Therefore, interpreting results should be carried out with caution, and further well-balanced prospective studies will be needed. However, the study found that habitual heavy drinking had a negative effect on skeletal muscle and fat, but not to the extent that complete abstinence from alcohol is necessary from the perspective of maintaining skeletal muscle mass or fat mass. Finally, we would like to emphasize that alcohol consumption is not only harmful in terms of maintaining skeletal muscle mass and fat mass, as long as the amount consumed is kept to a moderate level.

## 5. Conclusions

In Japanese SLD patients, drinking moderate amounts of alcohol does not adversely affect skeletal muscle mass or fat mass.

## Figures and Tables

**Table 1:** Baseline features.

	Men (n = 2435)	Women (n = 1207)	*p* Value
Age (years)	55.3 ± 10.9	56.8 ± 10.1	<0.0001
BMI (kg/m^2^)	25.5 ± 3.5	25.4 ± 4.0	0.2110
Group A/B/C/D	819/1147/125/344	749/354/36/68	<0.0001
Severity of fatty liver through US mild/moderate/severe	1150/975/310	643/437/127	0.0021
WC (cm)	90.2 ± 8.9	88.2 ± 9.6	<0.0001
Hemoglobin (g/dL)	15.1 ± 1.1	13.5 ± 1.1	<0.0001
ALT (IU/L)	32.2 ± 22.4	24.8 ± 17.0	<0.0001
GGT (IU/L)	56.2 ± 62.9	35.2 ± 41.7	<0.0001
Platelet count	25.0 ± 5.4	27.3 ± 6.3	<0.0001
Serum albumin (g/dL)	4.4 ± 0.2	4.3 ± 0.2	<0.0001
Triglyceride (mg/dL)	146.8 ± 105.1	119.3 ± 69.2	<0.0001
Fatty liver index	46.4 ± 25.0	36.1 ± 24.4	<0.0001
FIB4 index	1.15 ± 0.58	1.11 ± 0.48	0.5322
FBS (mg/dL)	100.2 ± 20.1	96.6 ± 17.0	<0.0001
Uric acid (mg/dL)	6.4 ± 1.3	5.3 ± 1.1	<0.0001
eGFR (mL/min/1.73 m^2^)	69.7 ± 13.2	72.0 ± 13.5	<0.0001
Systolic BP (mmHg)	126.2 ± 15.8	125.9 ± 17.2	0.1795
Diastolic BP (mmHg)	82.1 ± 11.6	77.3 ± 11.6	<0.0001
FF index (kg/m^2^)	19.0 ± 1.4	15.9 ± 0.9	<0.0001
F index (kg/m^2^)	6.4 ± 2.3	9.5 ± 3.2	<0.0001

Data are shown as a number or average (±standard deviation). BMI: body mass index, US: ultrasonography, WC: waist circumference, ALT: alanine aminotransferase, GGT: Gamma glutamyl transferase, FBS: fasting blood sugar, eGFR: estimated glomerular filtration rate, BP: blood pressure, FF index: fat-free mass divided by height squared, F index: fat mass divided by height squared.

**Table 2:** Results for comparison of baseline data other than FF index and F index among four types in men.

Men (n = 2435)	Group A (n = 819)	Group B (n = 1147)	Group C (n = 125)	Group D (n = 344)	Overall *p* Value
Age (years)	55.1 ± 11.6	54.9 ± 10.4	55.8 ± 11.6	56.7 ± 10.0	0.0226
BMI (kg/m^2^)	25.3 ± 2.8	26.0 ± 4.1	24.2 ± 2.2	24.6 ± 3.2	<0.0001
Severity of fatty liver on US, mild/moderate/severe	368/121/330	526/161/460	67/6/52	189/22/133	<0.0001
WC (cm)	89.6 ± 7.8	91.2 ± 9.9	87.6 ± 6.6	89.0 ± 8.3	<0.0001
Hemoglobin (g/dL)	15.1 ± 1.1	15.2 ± 1.0	15.1 ± 1.0	15.1 ± 1.1	0.2241
ALT (IU/L)	31.7 ± 19.6	33.1 ± 25.1	28.6 ± 17.0	31.7 ± 20.2	0.1300
GGT (IU/L)	40.0 ± 31.3	54.2 ± 55.4	59.8 ± 53.7	100.0 ± 109.1	<0.0001
Platelet count (/μL)	25.2 ± 5.4	25.1 ± 5.6	24.3 ± 5.4	24.5 ± 5.2	0.1458
Serum albumin (g/dL)	4.4 ± 0.2	4.4 ± 0.2	4.5 ± 0.3	4.4 ± 0.3	<0.0001
Triglyceride (mg/dL)	139.8 ± 104.1	144.6 ± 99.2	164.0 ± 112.5	164.7 ± 120.7	0.0172
Fatty liver index	41.9 ± 22.6	48.2 ± 26.0	43.5 ± 24.2	52.0 ± 25.9	<0.0001
FIB4 index	1.08 ± 0.58	1.13 ± 0.51	1.23 ± 0.61	1.35 ± 0.72	<0.0001
FBS (mg/dL)	98.0 ± 16.7	101.1 ± 21.6	100.9 ± 18.8	102.1 ± 22.5	0.0013
Uric acid (mg/dL)	6.2 ± 1.3	6.5 ± 1.2	6.4 ± 1.1	6.4 ± 1.3	<0.0001
eGFR (mL/min/1.73 m^2^)	68.8 ± 12.9	69.6 ± 13.1	71.5 ± 11.7	71.8 ± 14.2	0.0015
Systolic BP (mmHg)	123.6 ± 15.6	126.7 ± 15.4	126.1 ± 15.4	130.8 ± 16.8	<0.0001
Diastolic BP (mmHg)	79.8 ± 11.5	82.7 ± 11.4	82.3 ± 11.2	85.5 ± 11.7	<0.0001

Data are shown as a number or average (±standard deviation). BMI: body mass index, US: ultrasonography, WC: waist circumference, ALT: alanine aminotransferase, GGT: Gamma glutamyl transferase, FBS: fasting blood sugar, eGFR: estimated glomerular filtration rate, BP: blood pressure.

**Table 3:** Results for comparison of baseline data other than FF index and F index among four types in woman.

Women (n = 1207)	Group A (n = 749)	Group B (n = 354)	Group C (n = 36)	Group D (n = 68)	Overall *p* Value
Age (years)	56.2 ± 9.9	57.8 ± 9.9	52.5 ± 8.8	59.3 ± 12.0	0.0007
BMI (kg/m^2^)	25.9 ± 4.0	24.8 ± 3.6	24.9 ± 4.7	22.6 ± 3.8	<0.0001
Severity of fatty liver on US, mild/moderate/severe	375/93/281	204/27/123	21/5/10	43/2/23	0.0203
WC (cm)	89.1 ± 9.7	87.3 ± 9.1	87.2 ± 9.5	83.6 ± 8.7	<0.0001
Hemoglobin (g/dL)	13.5 ± 1.1	13.4 ± 1.0	13.3 ± 1.3	13.4 ± 1.3	0.5860
ALT (IU/L)	25.7 ± 18.2	23.8 ± 15.9	23.1 ± 9.6	21.3 ± 11.1	0.0952
GGT (IU/L)	32.1 ± 29.8	34.9 ± 34.6	38.7 ± 27.9	69.1 ± 116.0	<0.0001
Platelet count (/μL)	27.6 ± 6.4	26.9 ± 5.6	27.9 ± 6.9	27.0 ± 8.4	0.4337
Serum albumin (g/dL)	4.3 ± 0.2	4.3 ± 0.2	4.3 ± 0.2	4.3 ± 0.3	0.2766
Triglyceride (mg/dL)	118.2 ± 62.4	119.3 ± 58.7	126.4 ± 74.6	126.7 ± 147.2	0.7194
Fatty liver index	37.6 ± 24.9	34.0 ± 23.0	38.3 ± 27.8	29.9 ± 22.8	0.0183
FIB4 index	1.07 ± 0.45	1.14 ± 0.46	0.95 ± 0.31	1.47 ± 0.77	<0.0001
FBS (mg/dL)	97.1 ± 18.6	96.1 ± 14.1	94.8 ± 13.5	94.4 ± 13.5	0.4886
Uric acid (mg/dL)	5.2 ± 1.0	5.3 ± 1.1	5.3 ± 1.1	5.4 ± 1.1	0.5568
eGFR (mL/min/1.73 m^2^)	71.8 ± 13.3	71.3 ± 13.2	75.1 ± 13.3	75.6 ± 16.6	0.0463
Systolic BP (mmHg)	125.9 ± 16.8	125.0 ± 17.3	123.4 ± 18.4	131.5 ± 19.1	0.0289
Diastolic BP (mmHg)	77.6 ± 11.3	76.4 ± 11.5	79.7 ± 15.1	78.7 ± 13.3	0.1720

Data are shown as a number or average (±standard deviation). BMI: body mass index, US: ultrasonography, WC: waist circumference, ALT: alanine aminotransferase, GGT: Gamma glutamyl transferase, FBS: fasting blood sugar, eGFR: estimated glomerular filtration rate, BP: blood pressure.

## Data Availability

Data available on request due to restrictions, e.g., privacy or ethical.

## Abbreviations

TG: triglyceride, SLD: steatotic liver disease, MASLD: metabolic dysfunction associated steatotic liver disease, ALD: alcoholic liver disease, US: ultrasonography, OMPU: Osaka Medical and Pharmaceutical University, F: fat, FF: fat-free, FLI: fatty liver index, BMI: body mass index, WC: waist circumference, SD: standard deviation.
